# Supplementary material for: Polyethylene Glycol: The Future of Posttraumatic Nerve Repair? Systemic Review
Source: Int J Mol Sci. 2019 Mar 24;20(6):1478. doi: 10.3390/ijms20061478 (PMC6471459; doi:10.3390/ijms20061478)
Supplement: Supplementary file 1 [file ijms-20-01478-s001.pdf]

Table S1. Animal studies on polyethylene glycol therapy in treatment of peripheral nerve injuries. Abbreviations: PEG = Polyethylene glycol, CAPs = compound action potentials, CMAPs = compound muscle action potentials, FF = foot fault asymmetry test, SFI = Sciatic Functional Index, MB = Methylene Blue. Analysis carried out entirely in vitro (some axonal dye applications) not included in the table.

Animal model (type of nerve injury), number of animals enrolled	PEG therapy protocol	Methods of evaluation	Results	Reference, publication year
**Guinea pig (sciatic nerve crush injury) n = 20**	Experimental groups: PEG-treated, control. PEG group: subepineurial injection of PEG (Mr 1800 PEG 50%, by weight, in distilled water) for 2 minutes, then rinse away with Ringer's lactate.<br>Control groups: Krebs solution-treated group, a distilled water-treated group.	Electrophysiological recordings (CAPs, muscle contraction force, displacement of the hind foot)	Direct application after crush injury: within the first 30 minutes after treatment, 6/8 PEG-treated animals and 1/12 control animals exhibited recovery.<br>4-hour delay: 4/6 PEG-treated animals and 1/6 control animals exhibited recovery.	[39] 2002
**Rat (sciatic nerve crush injury) n = 77**	Experimental groups: melatonin, Krebs saline + Ca^{2+} + melatonin, methylprednisolone, Krebs saline, Krebs saline + Ca^{2+}. In each study group, the lesion site was rinsed with a different solution of the substances above. Then, PEG treatment (Mr 2000 PEG 50%, by weight, in distilled water for 3 minutes) was applied and washed with Krebs saline.	Electrophysiological recordings (CAPs)	Pre-PEG application of solutions enhanced with: melatonin, Krebs saline + Ca^{2+} + melatonin, Krebs saline + Ca^{2+} produced a significantly higher percentage of PEG fusions than Krebs saline separately, based on CAPs.	[62] 2004
**Rat (sciatic nerve cut injury**	Experimental groups: PEG-treated, fibrin glue. After suture-based nerve repair, PEG (DuraSeal) was applied on the lesion site in	Electrophysiological recordings (muscle contraction force),	There were no significant differences in muscle contraction force between groups 10 weeks after primary surgery. Histologic evaluation: significant	[48] 2009

**[transection with suture-based repair])**<br>**n = 29**	one group, and fibrin glue (Tisseel) in another group.	histological assessment of scar tissue	reduction in scar thickness in the PEGgroup, no significant differences in nerve diameter between groups.	
**Rat**<br>**(sciatic nerve crush injury and cut injury [transection without repair])**<br>**n = 40, both nerves used in each rat**	Experimental groups: PEG-treated groups (crush injury + PEG), control groups (crush and cut injury). PEG-crush injury group: application of Mr 2000 PEG 50%, by weight, in distilled water for 1.5 minutes on nicked epineurium.<br>Crush injury control group: distilled water application.	Electrophysiological recordings (CAPs), axonal dye diffusion, motor function evaluation: foot fault (FF) asymmetry test, Sciatic Functional Index (SFI)	Successful PEG fusion: crush injury 31/32 (postoperative CAPs ≥ 0.5 mV through the lesion site), no postoperative CAPs detectable in control groups (n = 53).<br>Dye diffusion across the lesion site was observed in 17/18 PEG-treated crushed nerves; no dye diffusion across the lesion in the control groups (n = 31).<br>Motor recovery: SFI and FF asymmetry score – PEG-crush animals performed significantly better at:24 hours (FF) and 3 weeks (SFI and FF) postoperation compared with crush group animals ($p < 0.05$). This tendency did not persist at further assessment time points (4–8 weeks).	[47]<br>2010
**Rat**<br>**(sciatic nerve crush injury and cut injury [transection with and without suture-based repair])**<br>**n = 300**	Experimental groups: cut injury without repair, cut injury with suture-based repair, cut injury without repair + MB (Methylene Blue), cut injury with suture-based repair + MB, cut injury without repair + PEG, cut injury with suture-based repair + PEG, cut injury with suture-based repair + MB + PEG, crush injury untreated, crush injury + MEL (melatonin), crush injury + MB,	Electrophysiological recordings (CAPs), motor function evaluation: foot fault (FF) asymmetry test, Sciatic Functional Index (SFI)	Successful PEG fusion in all PEG-treated groups (postoperative CAPs ≥ 0.5 mV through the lesion site). The greatest CAP recovery in cut injury with suture-based repair + MB + PEG group of any treatment group ($p < 0.001$). No postoperative CAPs detectable in the control groups.<br>Motor recovery: a cut injury with suture-based repair + MB + PEG group had better SFI and FF score at each data point (1–12 weeks) than other	[52]<br>2012

	crush injury + PEG, crush injury + MEL + PEG, crush injury + MB + PEG. Surgical field was irrigated with Krebs Ca^{2+} free saline in all groups. In MB and MEL groups, solutions were applied for 1–3 minutes. In PEG-treated groups, surgical field was rinsed with PEG Mr 5000 PEG 50%, by weight, in distilled water for 1.5–2 minutes.		experimental groups ($p < 0.01$), except for cut injury with suture-based repair + PEG group.	
**Rat (sciatic nerve cut injury -repair with autograft insertion) n = 20**	Experimental groups: PEG + autograft, control – autograft. Surgical field was irrigated with Plasma-lyte A® (Ca^{2+} free solution) in both groups. In PEG-treated group, coaption sites were irrigated with: 1% solution of MB in sterile water for 1 minute, PEG (Mr 3350 PEG 50%, by weight, in sterile water) for 1 minute. In the control group, coaption sites were irrigated with sterile water. Finally, the wound was rinsed with Ringer's lactate in both groups.	Electrophysiological recordings (CAPs), motor function evaluation: foot fall asymmetry test (FF), Sciatic Functional Index (SFI), histological nerve analysis	Successful PEG fusion in all PEG-treated animals (postoperative CAPs ≥ 0.5 mV through the lesion site) (n = 10). No CAPs detectable in the control group postoperatively (n = 10).<br>Motor recovery: 1 and 3 days postoperatively, PEG-treated group had significantly improved FF (1 d: $p < 0.05$, 3 d: $p < 0.001$) and SFI (1 d: $p < 0.001$, 3 d: $p < 0.01$) than the control group.<br>Nerve histology: in the distal nerve parts, there was a statistically significant higher number of sensory and motor axons in the PEG-treated group compared with the control group ($p = 0.0189$ and $p = 0.0032$, respectively).	[45] 2012
**Rat (sciatic nerve cut injury -repair with allograft insertion) n = 37**	Experimental groups: PEG + allograft, control – allograft. PEG treatment: application of 1% MB in sterile distilled water on coaption sites, epineurial sutures placement, PEG (Mr 3350 PEG 50%, by weight, in sterile water) for 1 minute,	Electrophysiological recordings (CAPs), motor function evaluation – Sciatic Functional Index (SFI),	Successful PEG fusion of both ends of the allograft (postoperative CAPs through graft conduction).<br>Motor recovery: 3 days and 1, 2, 6 weeks postoperatively, PEG-treated group had significantly improved SFI than the control group ($p < 0.05$).	[54] 2015

	flushing with Ringer's lactate. Control group underwent the same protocol, without PEG application.	histological nerve analysis	Nerve histology: 6 weeks after surgery, PEG-treated group had a significantly higher number of viable myelinated axons in the nerve part distal to the allograft than the control group ($p = 0.0034$).	
**Rat (sciatic nerve cut injury -repair with neural tube placement) n = 16**	Experimental groups: neural tube + PEG, control – neural tube. Surgical field was irrigated with Plasma-lyte A® in both groups, nerve ends were approximated and the ends of the neural tube were sutured to epineurium. Next, both groups received 1% MB in sterile distilled water through a slit in the neural tube. Then PEG-treated group: PEG (Mr 3350 PEG 50%, by weight, in sterile water) for 1 minute, control group – sterile water. Finally flushing with Ringer's lactate in both groups.	Electrophysiological recordings (CAPs), motor function evaluation: foot fault (FF) asymmetry test, histological nerve analysis	Successful PEG fusion of both nerve ends to neural tube (postoperative CAPs through conduction).<br>Motor recovery: 7, 14 and 21 days postoperatively, PEG-treated group had significantly improved FF than the control group ($p = 0.007$, $p = 0.001$, and $p = 0.006$, respectively). This tendency did not persist at further assessment time points (28, 35 days).<br>Nerve histology: 5–6 weeks after surgery, PEG-treated group had significantly higher axon count (CA-II and Choactase staining) in the nerve part distal to the nerve tube than the control group ($p = 0.027$ and $p = 0.049$, respectively).	[53] 2015
**Rat (sciatic nerve crush injury and cut injury [transection with suture-based repair]) n = 135**	Experimental groups: crush injury in Ca^{2+} free or Ca^{2+} containing saline, cut injury in Ca^{2+} free or Ca^{2+} containing saline, control groups. Crush injury groups were further divided and received solutions with combinations of the following substances: Protein kinase A inhibitor (PKI), protein kinase C isozyme η pseudosubstrate fragment (ηPSF), protein kinase C isozyme θ pseudosubstrate fragment (θPSF), and MB; before PEG fusion.	Electrophysiological recordings (CAPs), motor function evaluation – Sciatic Functional Index (SFI), histological nerve analysis	Successful PEG fusion in crush injury and cut injury in Ca^{2+} free saline, PEG-treated groups (postoperative CAPs through conduction). Unsuccessful PEG fusion in crush injury in Ca^{2+} containing saline, PEG-treated groups and control groups (no postoperative CAPs through conduction).<br>In PEG-treated cut injury in Ca^{2+} containing saline groups, immediate suture-based repair and PEG protocol did not restore CAPs conduction, but if nerve ends were flushed with, or trimmed in Ca^{2+}	[46] 2016

	PEG-treatment: application of 1% MB in double-distilled water on lesion site for 1–2 minutes, PEG (Mr 3350 PEG 50%, by weight, in sterile double distilled water) for 1–2 minutes, flushing with Ringer's lactate. Respective control groups underwent the same protocol, without PEG application.		free saline, sutured and PEG-treated, CAPs conduction was restored.<br>Motor recovery: no significant differences in SFI recovery between any crush injury groups. In cut injury groups:<br>• no significant differences between cut injury in Ca^{2+}-free and Ca^{2+}-containing saline without trimmed ends of PEG-treated groups and respective control groups<br>• significant differences in SFI recovery between cut injury in Ca^{2+}-free and Ca^{2+}-containing saline with trimmed ends of PEG-treated groups and respective control groups.<br>Nerve histology: 6 weeks after surgery, PEG-treated cut injury group had significantly lower mean axonal diameters than unoperated control ($p < 0.01$).	
**Rat (femoral nerve cut injury [transection with suture-based repair]) n = 20**	Experimental groups: PEG-treated, control. PEG treatment: application of 1% MB in sterile distilled water on coaption sites, epineurial sutures placement, PEG (Mr 3350 PEG 50%, by weight, in sterile water) for 1 minute, flushing with Ringer's lactate. Control group - only suture-based repair.	Axonal dye diffusion	8 weeks after surgery, PEG-treated group showed worse motor neuron reinnervation accuracy (preference for motor pathway) compared with the control group.	[64] 2016
**Rat (sciatic nerve cut injury [transection with**	Experimental groups: PEG-treated (standard application hand-held syringe), PEG-treated + device (application with the device), control. Surgical field was irrigated	Electrophysiological recordings (CAPs), motor function evaluation: foot fault	Successful PEG fusion in 13/18 animals in standard PEG application group and 15/18 animals in PEG + device group (postoperative CAPs conduction	[55] 2017

**suture-based repair])**<br>**n = 96**	with Plasma-lyte A® in all groups. PEG treatment: application of 1% MB in sterile distilled water on coaption sites, epineurial sutures placement, PEG (Mr and % in the solution not specified) for 1 minute, flushing with Ringer's lactate. Control group underwent the same protocol, without PEG application.	(FF) asymmetry test, Sciatic Functional Index (SFI), axonal dye diffusion, diffusion tensor imaging, histological nerve analysis	restoration). No CAPs detectable in the control group immediately post repair (n = 18).<br>Motor recovery:<br>• PEG application group: significantly improved SFI at all time points (3 days – 12 weeks postoperatively, $p < 0.05$), significantly improved FF (at all time points except 5 weeks postoperatively, $p < 0.05$) compared with the control group<br>• PEG + device group: significantly improved SFI and FF at all time points (3 days – 12 weeks postoperatively, $p < 0.01$) compared with the control group.<br>Dye diffusion across the lesion site was significantly higher in the standard PEG application group ($p < 0.05$) and PEG + device group ($p < 0.01$) compared with the control animals.<br>Diffusion tensor imaging: number of tracts travelling through repair site was significantly higher in the standard PEG application group ($p < 0.05$) and PEG + device group ($p < 0.01$) compared with the control animals.<br>Nerve histology: in the distal nerve parts, there was a significantly higher number of motor axons in the standard PEG application group ($p < 0.05$, 1 and 4 weeks postoperatively) and PEG + device group (p

			< 0.01, 1, 4, 12 weeks postoperatively) compared with the control group.	
**Rat (sciatic nerve cut injury [transection with suture-based repair]) n = 30**	Experimental groups: PEG-treated, control. Both groups were further divided depending on time from injury to repair: 1, 8, 24 hours. Surgical field was irrigated with Plasma-lyte A® in all groups. PEG treatment: application of 1% MB in sterile distilled water on coaption sites, epineurial sutures placement, PEG (Mr 3350 PEG 50%, by weight, in sterile water) for 1 minute, flushing with Ringer's lactate. Control group underwent the same protocol, without PEG application.	Electrophysiological recordings (CAPs), motor function evaluation – Sciatic Functional Index (SFI), histological nerve analysis	Successful PEG fusion at all time points in PEG-treated groups (post-repair CAPs restoration through the lesion site). No CAPs detectable in respective control.<br>Motor recovery: 3 and 7 days postoperatively, all PEG-treated groups had significantly improved SFI than the respective control group ($p < 0.05$).<br>Nerve histology: 7 days after surgery, PEG-treated groups had significantly higher axons counts in the nerve part distal to the repair site than the respective control groups ($p < 0.05$).	[56] 2017
**Rat (facial nerve [mandibular branch] cut injury [transection with suture-based repair]) n = 60**	Experimental groups: PEG-treated, control. Both groups were further divided depending on time from injury to repair: 24, 72 hours. PEG treatment: application of Krebs Ca^{2+} free saline + MB on coaption sites for 3 minutes, epineurial sutures placement, PEG (Mr 5000 PEG 50%, by weight in double-distilled water) for 2 minutes, flushing with Krebs Ca^{2+} containing saline for 3 minutes. Control group underwent only suture-based repair.	Electrophysiological recordings (CMAPs), histological nerve analysis	CMAPs recorded 6 weeks postoperatively:<br>• latency – lower in PEG-treated 72-hour-delay repair group compared with both control groups ($p < 0.01$)<br>• duration – lower in PEG-treated 24-hour-delay repair group, compared with both control groups and PEG-treated 72-hour-delay repair group ($p < 0.01$).<br>Nerve histology: 6 weeks after surgery, PEG-treated groups had significantly larger axonal diameters when compared with both control groups ($p < 0.001$). 21/45 animals died before study endpoint.	[57] 2018

**Rat (facial nerve cut injury [transection with suture-based repair]) n = 40**	Experimental groups: PEG-treated, control. Surgical field was irrigated with Plasma-lyte A® in all groups. PEG treatment: application of 1% MB in sterile distilled water on coaption sites, epineurial sutures placement, PEG (Mr 3350 PEG 50%, by weight, in sterile water) for 1–1.5 minutes, flushing with Ringer's lactate. Control group underwent the same protocol, without PEG application.	Motor function evaluation: eye blink reflex and vibrissae movement, axonal dye diffusion, histological muscle analysis	No significant differences between PEG-treated groups and simple suture-based repair groups in any measured parameter.	[60] 2018
**Rat (sciatic nerve cut injury [transection with suture-based repair]) n = 53**	Experimental groups: PEG-treated, control. Surgical field was irrigated with Plasma-lyte A® in all groups. PEG treatment: application of 1% MB in sterile distilled water on coaption sites, epineurial sutures placement, PEG (Mr 3350 PEG 50%, by weight, in distilled water) for 1–2 minutes, flushing with Ringer's lactate. Control group underwent the same protocol, without PEG application.	Electrophysiological recordings (CAPs, CMAPs), motor function evaluation - Sciatic Functional Index (SFI), histological nerve analysis	Successful PEG fusion in PEG-treated animals (postrepair CAPs and CMAPs restoration through the lesion site). Postoperatively, no CAPs and CMAPs detectable in the control group.<br>Motor recovery: 42 days postoperatively, PEG-treated group had significantly improved SFI compared with the control group ($p < 0.05$).<br>Nerve histology: PEG-treated nerves had significantly larger axonal and fiber diameters when compared with the control group at 21 and 42 days postoperatively ($p < 0.001$).	[58] 2018
**Rat (sciatic nerve cut injury –repair with autograft or allograft insertion)**	Experimental groups: PEG-treated, control. Both groups were further divided: autograft or allograft insertion. Surgical field was irrigated with Plasma-lyte A® in all groups. PEG treatment: application of 1% MB in sterile distilled water on coaption sites,	Electrophysiological recordings (CAPs, CMAPs), motor function evaluation – Sciatic Functional	Successful PEG fusion in PEG-treated animals (postrepair CAPs and CMAPs through conduction). No CAPs and CMAPs detectable in the control group immediately postrepair.	[59] 2018

**n = 79**	epineurial sutures placement, PEG (Mr 3350 PEG 50%, by weight, in distilled water) for 1–2 minutes, flushing with Ringer's lactate. Control group underwent the same protocol, without PEG application.	Index (SFI), histological nerve analysis	Motor recovery: 42 days postoperatively, PEG-treated group had significantly improved SFI compared with the control group ($p < 0.001$).<br>Nerve histology: PEG-treated nerves had significantly larger axons when compared with the control group at all harvest time points ($p < 0.001$).
